# On the rigidity of the preparation effect

**DOI:** 10.3389/fpsyg.2026.1787472

**Published:** 2026-06-26

**Authors:** Tomer Sahar, Tal Makovski

**Affiliations:** Department of Education and Psychology, The Open University of Israel, Ra’anana, Israel

**Keywords:** attention, boundary, distractor rejection, phasic alertness, temporal

## Abstract

The preparation effect (PE) describes enhanced attention and faster responses of dot-probes when stimuli are expected to appear. Prior work portrayed PE as a rigid, mandatory, process-all mechanism that boosts alertness for any upcoming event, largely insensitive to stimulus relevance, valence, or individual differences. The present study tested key boundary conditions of this effect across three experiments. In Experiment 1, we manipulated distractor probability and found a robust PE only under complete certainty (100% distractors), but not under a probabilistic context (50%), indicating that strong temporal expectations are required to trigger preparation. There was no difference between latencies of probe-dot detection under 25 and 75% distractor probability (Exp. 1b). Experiment 2 aimed at testing the PE across time, and distractor presence (0 or 100%) was manipulated between subjects. Dot-probe responses were consistently faster in the distractor group than in the no-distractor group, and this advantage remained stable across blocks, suggesting that the PE constitutes a durable alerting mode that, unlike other proactive effects, does not decay over time. Experiment 3 replaced the dot-probe onset detection with an offset-detection probe and found no significant RT benefit under this condition. Together, these findings demonstrate both the robustness and the limits of the PE. They also highlight the similarities and differences between the PE and other proactive control and phasic alertness effects, and call for a more nuanced explanation that considers both observers’ temporal expectations and probe demands.

## Introduction

How do observers prepare for the presentation of visual stimuli at a specific moment in time? Furthermore, is there a specific way in which the visual system prepares for distracting information, given its limited capacity and the need to prioritize visual information? The ability to attend to moments in time have been studied in tasks in temporal attention tasks (Coull and Nobre, 1998), typically by presenting a simple warning signal that precedes a target by some interval (e.g., Han and Proctor, 2022; Los et al., 2014). These studies have shown that when a particular moment reliably predicts the target’s onset, observers build up a readiness state that speeds their responses. This preparatory state reflects an active anticipation that the system deploys toward whenever an event is predicted to occur next, regardless of whether that event is task-relevant or not (Lawrence and Klein, 2013; Los et al., 2014). Importantly, a similar preparatory mechanism is engaged even when the observers expect a distractor to appear, merely by top-down knowledge and without a warning signal (Makovski and Chajut, 2021; Chen et al., 2023, 2026; Makovski, 2019; Lindzen et al., 2025; Shoval and Makovski, 2025).

The *Preparation Effect* (PE) refers to the effect of increased attention on a predicted moment in time, independent of whether the upcoming event is relevant, distracting, or irrelevant (Chen et al., 2026; Leclercq and Charras, 2026; Makovski, 2019; Shoval and Makovski, 2025). It has been shown that the effect is neither temporally nor spatially specific (Chen et al., 2026; Leclercq and Charras, 2026; Makovski, 2019) and therefore reflects an increase in general readiness or alertness to process upcoming stimuli. The effect was also not modulated by the distractor emotional content (Makovski and Chajut, 2021), and individual differences in working memory capacity or the ability to reject distractors (Lindzen et al., 2025), and hence it was suggested that in the face of any upcoming stimuli, even distracting information, the system operates in a “process-all” mode (i.e., mandatory non-selective attentional mechanism) which drives this effect.

**Figure 1:**
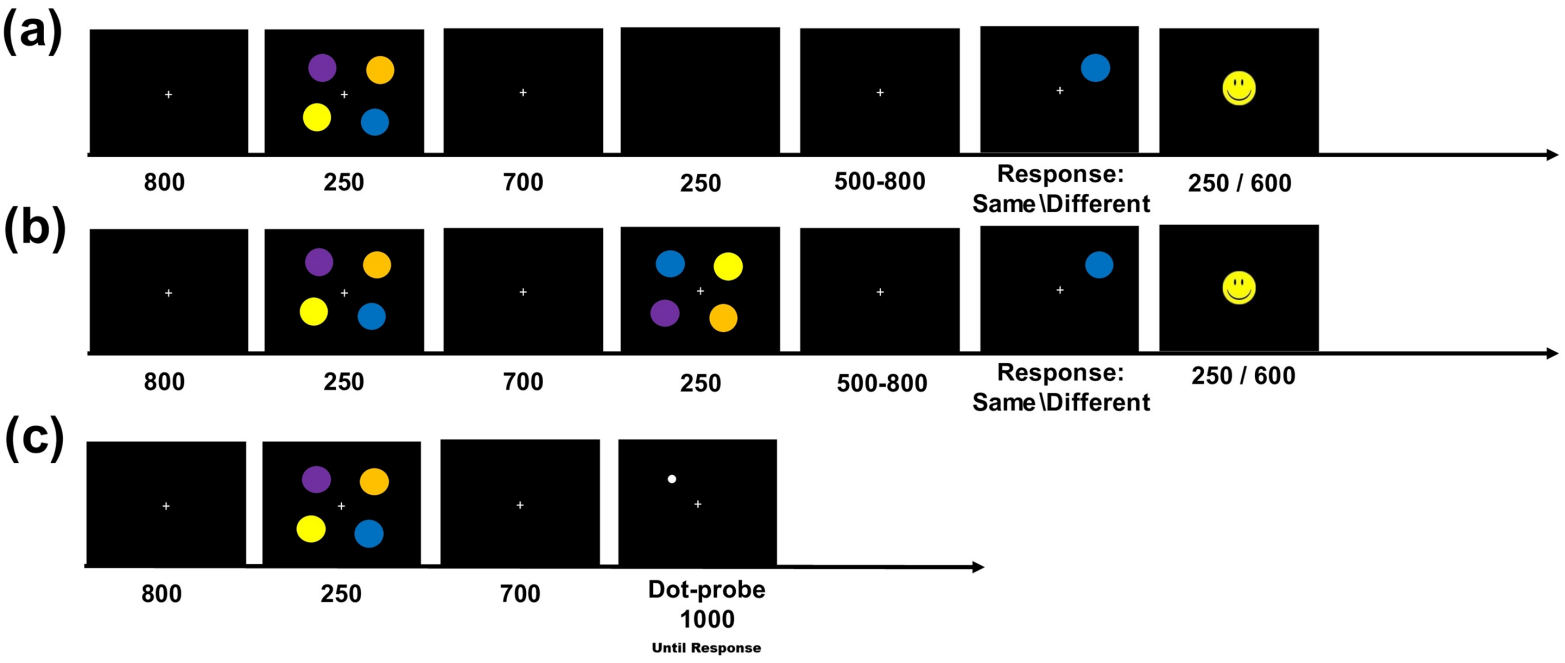
Schematic illustration of Experiment 1 trials: **(a)** No-distractor memory trials. **(b)** Distractor memory trials. **(c)** Dot-probe trials. Timing is shown in milliseconds. Experiments 1a and 1b examined different combinations of No-distractor and Distractor trials within a block, together with dot-probe trials.

Although the PE was found to be robust, it is important to consider the methodological limitations and the boundary conditions of the effect, as these conditions can illuminate its underlying mechanism and situate it within the broader context of preparatory processes. The core paradigm combines a visual short-term memory task with a speeded probe task as an index of attentional deployment (Makovski, 2019). As illustrated in Figure 1, on each trial, participants briefly encode a set of colored items and hold them in memory over a short delay. In Distractor blocks, a distracting display reliably appears during this delay at a fixed moment in time. In No-distractor blocks, no distracting information appears, but to match the temporal events of the trials, the fixation cross disappears for the same duration. Unexpectedly and infrequently, a small dot appears at the critical moment of the expected distractors, and participants are asked to press a key as quickly as possible upon detecting it. Because the dot probe appears at the exact moment distractors are expected, the speed of detection reflects how ready the observer is at that moment. Faster dot detection in Distractor blocks than in No-distractor blocks indicates that expecting a distractor heightened the observer’s general alertness at that moment, consistent with the notion of proactive facilitation rather than suppression.

The original study (Makovski, 2019) reported seven experiments using this paradigm. Across experiments, in which either a distractor or an informative display was expected, responses to the dot-probe were faster in blocks where additional stimuli were expected than in blocks where no stimulus was expected. This facilitation was observed even when the probe appeared at locations that never contained memory items or distractors, and even when the probe appeared before the expected stimuli. Notably, when the dot-probe appeared after the distractor’s appearance, there was no dot-probe advantage in distractor blocks, ruling out differences in general arousal as an explanation. Instead, Makovski (2019) suggested that the PE results from a nonspecific increase in phasic alertness that globally boosts readiness whenever a stimulus is about to appear, even if this stimulus is clearly irrelevant or harmful to the primary task.

Phasic alertness has often been studied using an informative warning signal before target presentation, and the typical finding is that the warning signal speeds responses by elevating general readiness (e.g., Hackley, 2009; Posner and Boies, 1971; Petersen and Posner, 2012; Schneider, 2019). Past studies have demonstrated that temporal warning signals that specify when something is about to appear produce alerting benefits. These benefits are typically expressed as faster RT, but sometimes at the expense of reduced accuracy, depending on the reliability of the cue and observers’ expectations (Han and Proctor, 2022, 2023; Lawrence and Klein, 2013). While the tasks examining external warning signals and PE are different, they share a common underlying mechanism of temporal preparation: in both paradigms, the observer uses learned temporal regularities (i.e., when events reliably occur) to boost nonspecific, general-purpose alerting readiness at the expected moment.

It seems, therefore, that the PE is an instance of a more general alerting mechanism: when an event is expected at a fixed time, the system enters a high-alert, process-all mode, boosting general readiness at the anticipated moment rather than selectively filtering or suppressing processing. In light of previous findings demonstrating the rigidity of the PE, the goal of the current study was to identify its boundary conditions (if any). That is, understanding when this preparatory response holds and when it might be attenuated or absent will allow us to better characterize the underlying mechanism of the PE and relate it to other temporal attention mechanisms.

Experiment 1 tested whether the preparation benefit in the PE paradigm is sensitive to the probability of an event occurring, as is the case with other phasic alertness phenomena (Los et al., 2014). Experiment 2 examined whether the PE holds even after participants gain extensive practice or whether it habituates over time, as was found in other preparatory paradigms (Cunningham and Egeth, 2016). Experiment 3 tested whether the PE benefit persists when the probe is defined by other stimulus attributes and not by an abrupt onset. That is, we asked whether a preparation benefit would emerge even when observers are asked to detect an offset event (i.e., “something disappearing”) instead of an onset event (i.e., “dot appearing”). This will clarify the role of attentional set (Folk et al., 1992) in modulating the effect and, more broadly, will address the question of whether phasic alertness is specifically tied to onset detection.

In sum, although the PE was found to be robust, it is important to consider its limitations and the boundary conditions of this effect. This would help clarify whether the PE reflects a general-purpose mechanism of attentional preparation that operates rigidly across a range of conditions, or whether it depends on specific contextual factors that may limit its generalizability.

## Experiment 1

One account of how temporal regularities shape alertness and temporal preparation (e.g., multiple trace theory, MTP; Los et al., 2014) suggests that each trial leaves an episodic memory trace that eventually forms inhibition when the expected event has not yet occurred or activation when the event occurs. Over many trials, these traces accumulate and are jointly retrieved to determine how prepared observers are at each point in time: moments that repeatedly host the expected event collect strong activation traces and therefore support fast, efficient responding, whereas moments that are repeatedly passed without an event collect inhibitory traces and remain associated with slower responses. Critically, the account predicts that robust preparation (at a particular moment) requires that the critical moment be reliable and consistent. That is, the expected event must occur on most trials. Naively, this account seems to suggest a gradual effect, based on the distractor’s occurrence history.

Experiment 1 tested whether the PE is sensitive to the frequency with which distraction is expected. Prior work that showed a robust PE always used complete certainty (either a block with distractors or without). If the PE reflects a default, “always-on” response of the alerting system to any temporally expected event, then even a relatively low probability of distraction should be sufficient to elicit robust preparation. Conversely, findings from probabilistic tasks (e.g., visual search and contingent-capture paradigms, Gaspelin and Luck, 2018; Theeuwes, 2010) and statistical learning (e.g., Duncan et al., 2025) suggest that proactive effects might scale with the likelihood of upcoming events (Geng, 2014; Wang et al., 2019). To address this question, Experiments 1a and 1b varied distractor probability within the same paradigm: Experiment 1a contrasted blocks without distractors, medium distractor probability (50%), and certain distraction (100%), whereas Experiment 1b focused on intermediate probabilities (25% vs. 75%).

### Method

#### Participants

Participants were between the ages of 18–40. All reported normal or corrected-to-normal vision and gave informed consent in accordance with the Open University of Israel Ethics board (approval #3308). Participants took part in the experiment for a course credit and were allowed to stop the experiment with no penalty. Based on a previous study (Shoval and Makovski, 2025), we calculated that a sample size of *N* = 50 provides at least 90% statistical power to detect a medium-sized effect (Cohen’s *d* = 0.5, alpha = 0.05) with a two-sided paired *t*-test. Participants only participated in one experiment; 50 participants completed Experiment 1a (14 Males, 36 Females, mean age = 25.6) and 48 participants completed Experiment 1b (14 Males, 34 Females, mean age = 26.56).

#### Stimuli

The experiments were programmed in MATLAB using Psychtoolbox-3 (Brainard, 1997; Pelli, 1997). Stimuli were presented on a standard PC with a 23.5″ LCD Eizo Foris monitor (1920 × 1,080 resolution, 120 Hz). Viewing distance was 57 cm in a dim, quiet room, and participants responded via a standard keyboard. The fixation cross was a white (255,255, 255) cross [20 × 20 pix]. The memory array consisted of 4 colored circles, sampled without replacement from a pool of nine colors: [Red (255,0,0), Green (0,255,0), Blue (0,0,255), White (255,255, 255), Yellow (255,255,0), Purple (127,0,127), Orange (255,140,0), Brown (115,58,0), Azure (0,0,255), radius = 35 pix]. The circles were placed equidistantly on an imaginary circle (radius = 150 pix) in the center of the screen. The dot was a white (255,255, 255) dot [25 × 25 pix], it appeared in one of the colored circles’ locations. All displays appeared on a black (0,0,0) background.

#### Procedure

All trials began with a fixation cross for 800 ms, followed by a memory array of four colored items for 250 ms, and a blank fixation display for 700 ms after memory encoding. In Distractor trials, a distracting display: a shuffled colors-location display appeared for 250 ms. To equate visual events, in the No-distractor trials, only the fixation cross disappeared at the expected time of the distracting display. After a random interval of either 500, 600, 700, or 800 ms, the memory test probe appeared. The test probe was one randomly chosen color-location from the encoding display. Participants were asked to report as accurately as possible, with no time limit, whether it was the “same” (press “S”) or “different” (press “D”) than the encoding display. “Same” and “different” probes were equally distributed across blocks. A happy face icon was presented for 250 ms after a correct response, while a sad face icon appeared for 600 ms after an incorrect response. There was a 200-ms blank screen between trials. In dot-probe trials, a small white dot appeared 700 ms after the memory array (i.e., at the expected timing of the distractor) at the location of one of the colored circles. Participants were asked to press the space bar as fast as they could upon detection of the dot. The dot remained visible for 1,500 ms or until a response was registered. If the response time exceeded 1,000 ms, a feedback text “Too slow” appeared for 800 ms. There was no memory test, and the memory probe did not appear in the Dot-probe trials. Dot-probe trials randomly appeared in only 25% of trials of each block; they did not appear in succession, nor immediately after a break.

Experiment 1a manipulated the number of distracting trials in each block (i.e., 0, 50, or 100% of Distractor trials). There were 3 blocks, each block with 120 trials (for a total of 360 trials). Each block contained 30 trials Dot-probe trials (25% from the block’s trials) and 90 memory trials (75% from the block’s trials). Distractor block order was randomly determined across the six possible combinations.

Experiment 1b manipulated only the intermediate probability of distracting trials (i.e., 25 or 75% of the Distractor trials within each block). There were two blocks, each with 120 trials (for a total of 240 trials). Again, each block contained 30 dot trials (25%) and 90 memory trials (75%). Distractor block order was counterbalanced across participants.

Participants performed 32 practice trials before the experiment (a No-distractor block followed by a Distractor block, each with 14 memory trials and 2 dot-probe trials). In both experiments, participants were notified at the beginning of each block whether distractors would appear and their proportion of the trials (e.g., half of the trials, all of the trials, etc.). Figure 1 depicts an illustration of the trials’ structure.

### Statistical analysis

The results were analyzed using JASP Team, (2021 version 19.1). When applicable, a Greenhouse–Geisser sphericity correction was applied. All multiple comparisons were Bonferroni corrected. For some variables that violated normality (Shapiro–Wilk test), a Wilcoxon test or a Conover’s non-parametric post-hoc test was applied. Since both parametric and non-parametric tests converged on the same conclusions, and for the sake of clarity, we report the uncorrected tests (i.e., ANOVA with partial eta squared and Student’s *t*-test with Cohen’s *d*) and degrees of freedom. Complete analyses available at https://osf.io/8a3ec.

#### Data exclusion

We excluded from the analyses participants who scored less than 50% correct overall or in the No-distractor condition. We also excluded participants who did not respond to 50% or more of the dot-probe trials. This resulted in valid data from 44 participants for Experiment 1a and 43 participants for Experiment 1b. The exclusion of these participants did not change any of the conclusions.

### Results

**Figure 2:**
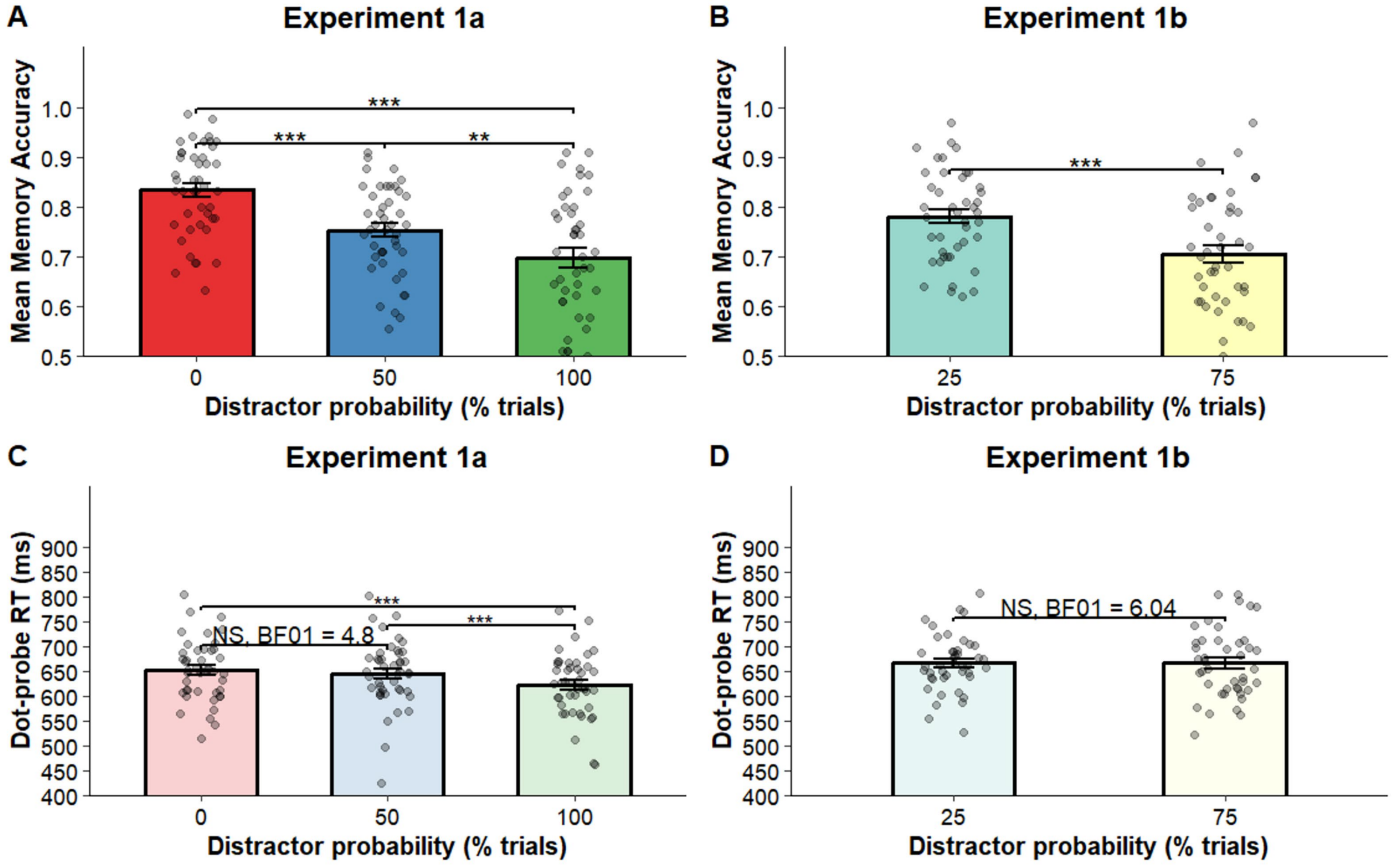
Experiment 1 results: **(A)** Accuracy data of Experiment 1a memory trials as a function of distractor probability. **(B)** Accuracy data of Experiment 1b memory trials as a function of distractor probability. **(C)** Mean RT of Experiment 1a dot-probe trials as a function of distractor probability. **(D)** Mean RT of Experiment 1b dot-probe trials as a function of distractor probability. Black dots represent individual scores. ** denotes *p* < 0.01, ^***^ denotes *p* < 0.001. BF_01_ denotes the Bayes factor in favor of the null hypothesis of no difference. Error bars represent 1 SEM.

Figure 2 shows a summary of Experiments 1a and 1b results.

*Exp. 1a Memory accuracy.* Accuracy declined as distraction probability increased, *F*(2, 86) = 43.89, *p* < 0.001, $\eta_{p}^{2}$ = 0.505. No Distraction – 0%: *M* = 0.835, SD = 0.090. 50%: *M* = 0.754, SD = 0.090. 100%: *M* = 0.698, SD = 0.126. Bonferroni corrected Post-hoc comparisons showed significant difference between conditions; 0–50%: *t*(43) = 6.8, *p* < 0.001, *d* = 0.783, 50–100%: *t*(43) = 3.6, *p* = 0.002, *d* = 0.537, 0–100%: *t*(43) = 8.2, *p* < 0.001, *d* = 1.32.

For completeness, memory task response time (only correct responses) showed an increase with increased distractor probability: *F*(2, 86) = 7.251, *p* = 0.001, $\eta_{p}^{2}$ = 0.155, 0%: *M* = 1,371 ms, SD = 311, 50%: *M* = 1,480 ms, SD = 393. 100%: *M* = 1,502 ms, SD = 365. Post-hoc comparisons showed a significant difference for 0–100%: *t*(43) = 4.08, *p* < 0.001, *d* = 0.372. 0–50%: *t*(43) = 3.19, *p* = 0.008, *d* = 0.318, but not 50–100%: *t*(43) = 0.433, *p* > 0.910.

*Exp. 1a Dot-probe RT.* RTs decreased with higher distraction probability, *F*(2, 86) = 9.12, *p* < 0.001, $\eta_{p}^{2}$ = 0.175: No Distraction = 652.8 ms, 50% = 646.6 ms, 100% = 622.9 ms. Bonferroni corrected Post-hoc comparisons showed that dot trials on 100% distractor trials were significantly faster than dot trials in the 0%: Mean difference = 29.8 ms, *p* < 0.001, *d* = 0.466, and 50% distractor trials: Mean difference = 23.6 ms, *p* < 0.001, *d* = 0.369. Dot trials RT under 0 and 50% distractor trials were reliably not different: Mean difference = 6.2 ms, *p* > 0.900, Cohen’s *d* = 4.8 (Standard prior).

*Exp. 1b Memory accuracy.* Like Experiment 1a, performance decreased with distraction, *F*(1, 42) = 17.54, *p* < 0.001, $\eta_{p}^{2}$ = 0.295. Accuracy was higher at 25%, *M* = 0.781, SD = 0.092, than at 75% distraction, *M* = 0.705, SD = 0.117, *t*(42) = 4.1, *p* < 0.001, *d* = 0.726. Memory task response time (only correct responses) was slower when distractor probability increased: *t*(42) = 2.67, *p* = 0.011, *d* = 0.408, 25%: *M* = 1,447 ms, SD = 323, 75%: *M* = 1,521 ms, SD = 275.

*Exp. 1b Dot-probe RT.* There was no significant difference in the dot-probe RT between 25 and 75% distractor blocks, *t*(42) < 1, *p* > 0.900, BF_01_ = 6.0.

### Discussion

The results of Experiment 1 showed that the PE occurred only under complete certainty that distractors would appear, and it was not modulated by the distractor’s expected frequency. This finding situates the PE as contingent on a stable expectation that “something will happen” rather than on a mere increase in distractor likelihood. This pattern still links PE to phasic alertness triggered by strong temporal expectations about upcoming events, yet it appears more conditional than suggested by the “mandatory process-all” mechanism (see also Chen et al., 2023, 2026). Furthermore, no graded effect was found across the intermediate probability, as the MTP account (e.g., multiple trace theory, Los et al., 2014) might suggest. Yet, due to the episodic nature of the MTP account, a subtle graded effect might require more trials to accumulate. Importantly, the results indicate that the PE is reliable but requires strong and stable expectations.

Finally, note that Experiment 1b did not include a no-distractor block, and hence, one cannot be certain about the lack of PE in this experiment. Yet, given its absence in the 50% condition of Experiment 1a and the lack of any difference between the 25 and 75% conditions, it seems reasonable to conclude that PE was only expressed under 100% distractor probability.

## Experiment 2

Experiment 2 was designed to test the stability of the PE over time - does it diminish or habituate with prolonged exposure to expected distraction trials? Phasic alertness is typically characterized as a brief, cue-locked boost in responsiveness that often habituates when cues or events are repeated over time. This raised the question of whether PE reflects a transient surge in alertness that fades with time.

Foreperiod-based preparation can itself be remarkably stable over blocks, indicating that the alerting system can sustain elevated readiness when contingencies remain reliable (Han and Proctor, 2023). Thus, sustained PE might reflect an elevation of baseline readiness that is maintained as long as the context justifies it. This pattern of a non-attenuating preparation effect would therefore fit in a broader picture of sustained temporal preparation. In contrast, it was found that the “attentional white bear” phenomenon, in which observers are allocating attention in advance to expected distractor locations (Tsal and Makovski, 2006), or features (e.g., color, Moher and Egeth, 2012), can be reversed after extended practice with the stimuli (Cunningham and Egeth, 2016). Thus, the prolonged task allows us to examine the nature of alertness in PE.

To address this issue, participants were randomly assigned to a between-subjects Distractor condition (100% distractor vs. no-distractor blocks) and performed a long series of change-detection and dot-probe trials, allowing PE to be tracked across multiple blocks.

### Method

The trials’ structure was identical to Experiment 1, except that we used a between-subjects design to examine a prolonged task. All trials began with a fixation cross for 800 ms, followed by a memory array of four colored items for 250 ms, and a blank fixation display for 700 ms after memory encoding. In the Distractor block, a distracting display: a shuffled colors-location display appeared for 250 ms, in the No-distractor block, only the fixation cross disappeared at the expected time of the distracting display. After an interval of either 500, 600, 700, or 800 ms (constant within a block and in random block order^1^), the memory test probe appeared. All other events were identical to Experiment 1a. There were 4 blocks, each with 120 trials (for a total of 480). Each block contained 25% dot trials (30 trials) and 75% memory trials (90 trials). Participants were randomly and equally assigned to either the Distractor group or the No-distractor group. All the memory trials across the four blocks were according to group assignment. Participants performed 32 practice trials according to their group assignment (24 memory trials + 8 dot-probe trials).

To increase motivation in this experiment, participants received monetary compensation for their participation (~ $22). Power analysis showed that a sample size of 30 participants in each group, provides 90% power to detect a medium sized effect (*f* = 0.35) over 4 time points (*k* = 4, alpha = 0.05). However, only 28 participants completed the experiment in each group (No-Distractor: Females = 21, Males = 7, Mean age = 26.6. Distractor: Females = 21, Males = 7, Mean age = 27.1).

### Statistical analysis

The data from 56 participants were submitted to mixed Repeated Measures ANOVAs with Block as a within-subjects factor and the distractor group as a between-subjects factor. For clarity, we report the ANOVA, Student’s *t*-test (Bonferroni-corrected), and Cohen’s *d* effect size.

### Results

*Memory accuracy.* Memory accuracy improved significantly over time, *F*(3, 162) = 26.34, *p* < 0.001, $\eta_{p}^{2}$ = 0.328. Post-hoc (Bonferroni corrected) comparisons showed that accuracy in blocks 2, 3, and 4 was significantly higher compared to the first block (all *t*’s > 5, all *p*’s < 0.001). Overall memory accuracy was not significantly different between distractor groups, Distractors: *M* = 0.836, SD = 0.114, No-distractors: *M* = 0.862, SD = 0.099, *F*(1, 54) = 0.850, *p* = 0.361, BF_01_ = 2.6. In addition, the overall improvement across time was not different between the distractor groups, *F*(3, 162) = 0.258, *p* = 0.855.

RT of correct memory responses also showed improvement over time, *F*(3, 162) = 48.2, *p* < 0.001, $\eta_{p}^{2}$ = 0.472 (Mean RT’s of Blocks 1–4: 1247 ms, 1,257 ms, 1,168 ms, 1,130 ms) but did not show difference between groups, Distractor: *M* = 1,296 ms, SD = 209, No-distractor: *M* = 1,152 ms, SD = 436, *F*(1, 54) = 2.53, *p* = 0.117, BF_01_ = 1.3, nor any interaction, *F*(3, 162) = 1.705, *p* = 0.168.

**Figure 3:**
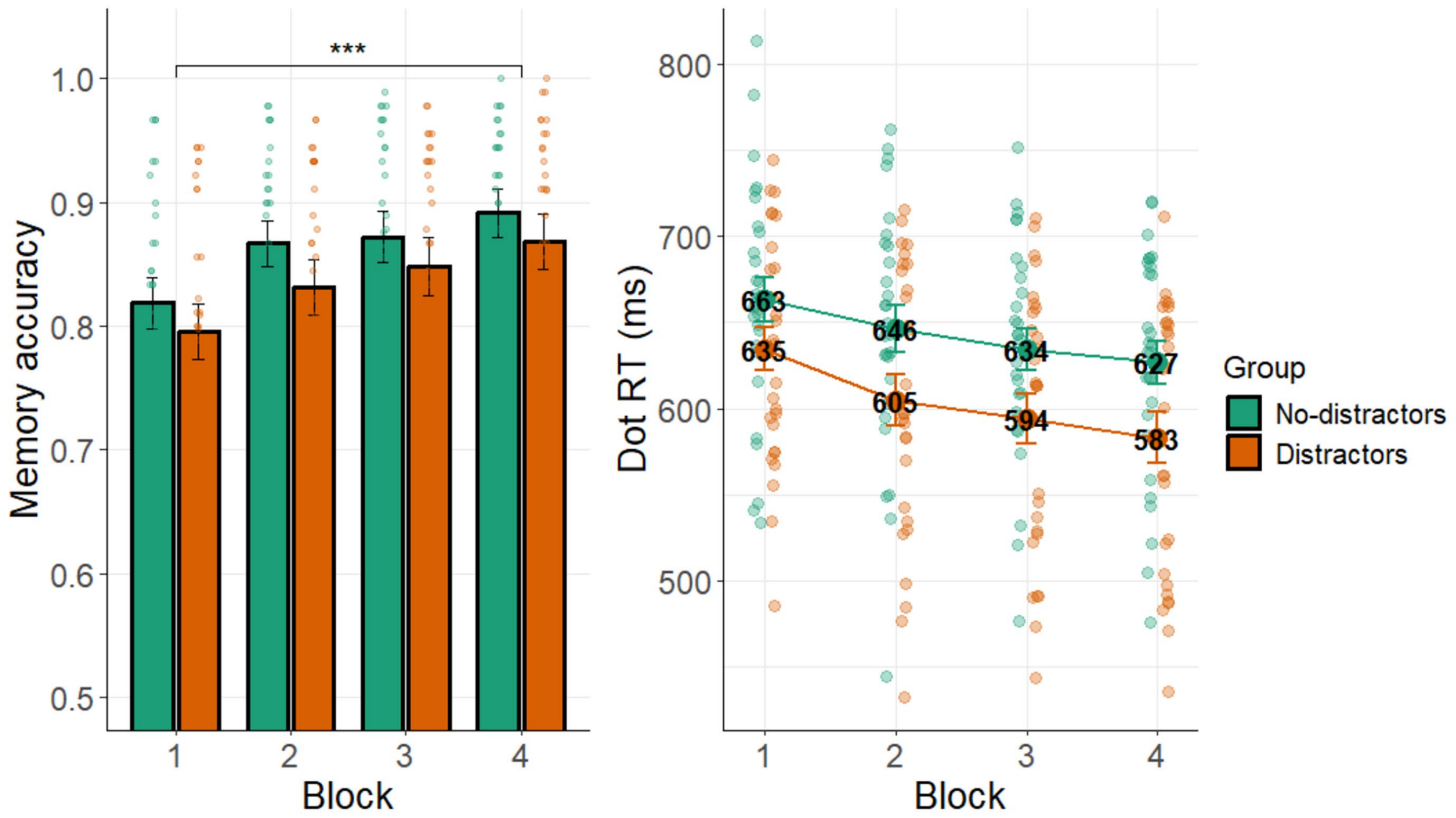
Results of Experiment 2. Left: Correct responses as a function of experimental block. Right: Dot-probe detection Response-time (RT) across blocks. Mean RT is shown in ms. Light points represent individual scores, ^***^ denotes *p*-value < 0.001. Error bars represent 1 SEM.

*Dot-probe RT.* There was a main effect of distractor condition, *F*(1, 54) = 4.65, *p* = 0.035, $\eta_{p}^{2}$ = 0.079. Dot-probe RT was faster for the Distractor group (*M* = 604 ms, SD = 70) than the No-distractor group (*M* = 642 ms, SD = 61). There was a significant effect of improvement across time as RTs decreased across blocks, *F*(3, 162) = 22.2, *p* < 0.001, $\eta_{p}^{2}$ = 0.291. Most importantly, the rate of improvement with time was similar between groups as there was no significant interaction between the blocks and Distractor condition, *F*(3, 162) = 0.685, *p* = 0.563. Figure 3 shows a summary of Experiment 2’s results.

### Discussion

In the dot-probe task, the Distractor group was consistently faster in detecting the dot-probe than the No-distractor group, replicating once again, even with a between-subjects design, the standard PE effect (Makovski, 2019). Importantly, there was no significant difference in the magnitude of the effect across time. It did not habituate or diminish over time.

The temporal stability of the preparation effect might seem, at first glance, to be a tonic alertness process. Tonic (or intrinsic) alertness reflects a sustained, slowly varying readiness that operates independently of discrete external cues and is maintained without external refreshment (e.g., Sturm and Willmes, 2001). Phasic alertness reflects a short-lived boost in processing speed triggered by an external warning signal that is sensitive to task factors and relevance (Lin and Lu, 2016). Critically, repeated phasic exposure does not accumulate into an elevated baseline state over time (Dupont et al., 2026).

The non-habituating, block-stable preparation effect seems consistent with a tonic profile; once distractor expectancy is established, it appears to be maintained as an enduring state. However, tonic elevation would predict a general rise in baseline processing, for instance, in faster memory-probe RTs or reduced overall errors, yet these patterns were not observed in Experiment 2 (nor in any of the previous PE studies). Instead, overall performance in the main memory task, as well as probe-dot detection latencies, improved similarly and at the same rate in both groups. Moreover, the PE has been shown to operate as a general boost in visual processing rather than a global enhancement of vigilance (Shoval and Makovski, 2025). Thus, it aligns with similar findings showing that phasic alertness can be a stable and non-habituating process over time (e.g., Han and Proctor, 2023; Weinbach and Henik, 2011; Tsal and Makovski, 2006).

Taken together, Experiment 2’s findings support the notion that the PE reflects time-locked readiness for the anticipated distractor rather than a broad elevation of arousal or tonic alertness. This pattern may arise from an attentional set that, once formed through distractor expectancy, operates as a sustained preparatory state without requiring the baseline arousal elevation characteristic of traditional tonic alertness.

## Experiment 3

Experiment 3 was designed to test the PE in a version of the paradigm that altered a core structural feature of the task: the nature of the dot-probe task used to index attentional deployment. In the standard dot-probe task, both the memory display and the probe are abrupt onsets, raising the possibility that faster probe responses are closely linked to an onset-biased attentional set (Folk et al., 1992).

Attentional sets reflect the top-down configuration of the visual system toward anticipated stimulus features or events, and they can be driven by both endogenous goals and exogenous task demands (Lawrence and Klein, 2013). A key question, therefore, is whether the increased phasic alertness underlying the PE reflects a narrow attentional set that is specific to the detection mechanism used to index attention, or whether it can also be captured by an offset-detection task.

Hence, Experiment 3 replaced the onset probe with an infrequent offset-detection event to test whether offset probes are sensitive enough to detect an increase in phasic alertness as manifested by the PE. Onset and offset changes probe attention through fundamentally different routes: onsets trigger automatic, bottom-up capture driven by the appearance of a new object (Yantis and Jonides, 1984), whereas offsets engage later visual processes, specifically memory and object-file updating, that require revising an existing object representation rather than registering a new one (Carmel and Lamy, 2014, 2015; Lamy et al., 2015). Offset detection is more resource-demanding and may rely more on endogenous than exogenous attentional mechanisms (Lawrence and Klein, 2013), making it a stronger test of whether the PE operates as a broad attentional set or as a response specifically tied to onset-based probe detection.

To assess the extent to which the PE can be detected by offset tasks, we modified a version of the basic PE paradigm (Makovski, 2019) to accommodate an offset probe task with a large sample size. To that end, unlike Experiments 1 and 2, Experiment 3 adapted a previous online version of the task (Lindzen et al., 2025).

### Method

#### Participants

We followed a previous online study (Lindzen et al., 2025, Exp.2, *n* = 153, Cohen’s *d* = 0.29), and calculated that a sample size of 150 provides 99% statistical power for detecting an effect sized 0.3 (Cohen’s *d*) with a paired samples two-sided *t*-test (alpha = 0.05, “pwr” package, Champely, 2006). We therefore recruited 150 participants through prolific, an online experiments platform (prolific.co). Participants were paid ~ 2.5 ₤ for participating, and were pre-screened by: Age 18–40, English as a first language, Vision Normal or corrected-to-Normal. Normal color vision, no ADD/ADHD, at least 10 previous submissions with an above 90% approval rate. Median time to complete the task = 16.36 min, Mean age = 29.2, Females = 98, Males = 52.

#### Procedure

**Figure 4:**
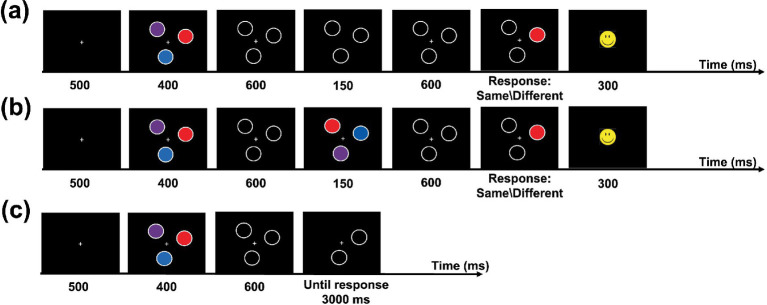
Schematic illustration of Experiment 3 trials: **(a)** No-Distractor memory trials. **(b)** Distractor memory trials. **(c)** Probe-offset trials: one of the colors’ placeholders disappeared (at the exact time the distractor would appear in a distractor trial), participants were asked to press the spacebar key as fast as they could.

The study ran online using Psychopy (Peirce et al., 2019) and Pavlovia (pavlovia.org). The basic paradigm was similar to the previous experiments, and mainly to Lindzen et al. (2025, Exp 1-2). Each trial started with a white fixation cross (0.01 × 0.01, normalized screen height, 10×10 pix in 1920 × 1,080 screen) for 500 ms. The encoding display was shown for 400 ms and consisted of three colored circles (0.08 × 0.08) randomly sampled from 10 colors list: Dark-red (RGB = 242, 63, 0), Light-green (RGB = 12, 242, 38), Yellow (RGB = 229, 229, 51), Bright violet (RGB = 63, 0, 229), Magenta (RGB = 255, 0, 204), Deep blue (RGB = 0, 191, 255), Orange (RGB = 242, 165, 25), Aqua (RGB = 25, 255, 153), Dark-green (RGB = 153, 204, 6), Cyan (RGB = 6, 216, 216). Figure 4 depicts the trial’s design.

On each trial, three circles, all with a white outline (width = 2 pix), were randomly placed (0.15 screen units) from the center and evenly separated. This was followed by a “place-holders” display, where only the white outline without colored fill remained on the screen for 600 ms. Trials’ design was similar to Experiments 1 and 2. All trials began with a fixation cross for 500 ms, followed by a memory array of 3 colored items for 300 ms, and a blank fixation display for 600 ms after memory encoding. In the Distractors block (Figure 4b), a distracting display: a shuffled colors-location display appeared for 250 ms., in the No-distractors block (Figure 4a) only the fixation cross disappeared at the expected time of the distracting display. In all displays the white outline without colored fill remained on the screen. After a fixed interval of 600 ms, the memory test probe appeared; one color from the encoding display either appeared at the same location as in the encoding display (“same” response) or at a different location (“different” response. Same and Different memory responses were evenly distributed across the block. Participants were asked to respond with a keyboard press (“s,”“d”) whether the color-location was the same or different. There was no time limit for response and accuracy was emphasized. After the response, a feedback display (a sad face icon for an incorrect response and a happy face icon for a correct response) appeared for 300 ms. Participants were encouraged to ignore the distracting display and were told at the start of each block whether the distractors would appear or not).

Importantly, there were two blocks of conditions: with distractors (100%) and No-distractors (0%). On each block, there were 30 randomly interleaved offset trials (Figure 4c). Offset trials timing matched that of the memory trials with one exception: at the moment the fixation cross disappeared (No-distractor trials) or distractors appeared (Distractor trials), one unfilled placeholder vanished, and participants were asked to respond with a spacebar press as fast as possible when they detected this event. The offset circle was randomly selected across trials. The other two placeholders remained visible on the screen, and if no response was registered within 3,000 ms, a “TOO SLOW!” text appeared for 300 ms. Each block (Distractor, No-distractor) had 90 memory trials and 30 probe-offset trials with the same restrictions as before. Participants completed both condition blocks with their order counterbalanced across participants. Participants completed 16 practice trials before the main task started (No-distractor: 6 memory + 2 offset trials, Distractor: 6 memory + 2 offset trials).

#### Statistical analysis

Data were analyzed using paired samples *t*-tests.

### Data exclusion

The data from 12 participants were removed (8 for overall responses below 50% correct, 4 for missing more than 50% of the offset trials).

#### Results

The memory accuracy was significantly better in No-distractor trials (*M* = 0.826, SD = 0.084) than Distractor trials (*M* = 0.74, SD = 0.13), *t*(137) = 9.35, *p* < 0.001, Cohen’s *d* = 0.796. Response times of correct memory responses were not different between the conditions, *t*(137) = 0.777, *p* = 0.438, BF_01_ = 7.8, Distractor: *M* = 1,053 ms, SD = 276. No-distractor: *M* = 1,023 ms, SD = 382.

Unlike the dot-probe trials of Experiments 1 and 2, there was no significant difference in the offset-probe RT between distractor conditions, Distractor: *M* = 778 ms, SD = 143; No-distractor: *M* = 792 ms, SD = 154, *t*(137) = 1.71, *p* = 0.088, BF_01_ = 2.5, suggesting that PE was not established.

We also compared the offset RTs with the dot-probe RTs from Lindzen et al.’s data (Exp.2, 2025, OSF). Due to methodological differences in timing, this is a rough comparison for context, not a strict generalization test. We submitted these data to a mixed ANOVA with distractor condition as a within-subject factor (Distractor, No-distractor) and Experiment as a between-subjects factor (Offset vs. Onset). There was a main effect of distractor, as detection RTs were faster under distraction (*M* = 705 ms vs. 728 ms), *F*(1,236) = 18.5, *p* < 0.001, $\eta_{p}^{2}$ = 0.073. The between-subjects factor of Experiment was also significant. Offset detection (*M* = 785 ms) was slower by about 137 ms compared to the dot-probe, *M* = 648 ms, *t*(236) = 8.92, *p* < 0.001, *d* = 1.1. The interaction between Distractors and Experiments was not significant, *F*(1, 236) = 2.85, *p* = 0.093, BF_01_ *=* 0.654. This analysis showed a main effect of distractor condition without a significant interaction; therefore, it does not statistically confirm the lack of PE for offsets.

#### Discussion

Experiment 3 failed to find a significant facilitation of offset detection in a highly powered experiment. The results did show a numerical (but insignificant) trend: RT in the Distractor condition was faster by 14 ms than in the No-distractor condition (which is hardly comparable to the expected effect of at least ~ 40 ms). The follow-up between experiment comparison did not detect a significant interaction between this experiment and a previous study with a similar design. Yet, the overall slower responses in the present experiment and the between-subjects analysis (coupled with a few methodological differences) might conceal or diminish any effect. Thus, more research is needed in order to better compare the magnitude of the onset and (possible) offset effects. Still, the highly powered (and direct) offset Experiment 3 did not show a reliable and significant facilitation benefit, in contrast to the consistently replicated onset-based effects.

## General discussion

Previous studies characterized the preparation effect (PE) as a rigid, largely mandatory mechanism that boosts alertness and promotes the encoding of any upcoming information, regardless of its relevance, whenever a stimulus is reliably expected (Makovski, 2019; Makovski and Chajut, 2021; Shoval and Makovski, 2025; Lindzen et al., 2025). The existing findings suggested a broad non-specific increase in attentional deployment, such that both distracting and non-distracting events benefit similarly from this preparatory state. The present study probed the rigidity of this effect and its boundary conditions by examining three factors: the probability of distractors, the stability of preparation over an extended time, and the sensitivity of preparation to the type of probe event detected.

Experiment 1a showed that a preparation advantage in dot-probe detection emerged only when distractors were guaranteed (100% distractor trials) and not when they were probabilistic (50% distractor trials). Experiment 1b found no difference between 75 and 25% distractor blocks. Together, Experiments 1a and 1b established that the PE does not operate under probabilistic conditions and requires complete certainty that a distractor will appear.

The multiple trace theory of temporal preparation (Los et al., 2014) suggests that each trial creates a memory trace. Over trials, these traces jointly determine the readiness temporal profile: a reliable event collects strong activation traces, whereas an event that is only sometimes followed by another event yields weaker traces. A 100% distractor block provides a maximally reliable critical moment, whereas 25, 50, and 75% blocks generate a mixture of activating and inhibitory traces that are collectively (either by weak signal or too few trials) insufficient to produce a robust preparatory advantage.

This probability boundary of PE parallels findings from other preparatory paradigms. For example, Han and Proctor (2023) showed that enhanced information processing by a warning signal critically depends on foreperiod constancy within a block: a fixed, predictable foreperiod yields a reliable RT advantage, whereas variable or uncertain foreperiods weaken and destabilize preparation (Han and Proctor, 2022; Los et al., 2014). The present results show an analogous pattern: certainty about temporal occurrence enables preparation, whereas partial probability does not.

This finding also sets the PE apart from other preparatory mechanisms, particularly proactive suppression. Proactive suppression of anticipated distractors can be acquired incrementally from graded statistical regularities tasks, which involve a selective reduction of interference, strongly shaped by expectations, and adapts flexibly to the statistical structure of the environment (Duncan et al., 2025; Gaspelin and Luck, 2018; van Moorselaar and Slagter, 2019; Theeuwes, 2010). The PE does not follow this graded pattern. Even at 75% distractor probability, no preparatory advantage was observed, suggesting that the PE and proactive suppression operate through fundamentally different mechanisms.

A recent study (Leclercq and Charras, 2026) tested this contrast directly using both a dot-probe detection task and a foreperiod manipulation. Dot-probe detection remained facilitated regardless of whether distractors were interfering or non-interfering, and regardless of foreperiod duration or distribution. Temporal preparation supported by longer foreperiods improved dot-probe detection, but this facilitation did not translate into suppression of the anticipated distractors. The PE and proactive suppression thus qualitatively differ: temporal certainty is required for alerting and facilitation, even when conditions would seemingly favor suppression.

These findings further rule out an alternative explanation arguing that the fixed timing of the distractors renders them task-relevant to the dot-probe task, by serving as a temporal anchor. A temporal-anchor account predicts graded facilitation with distractor probability, yet no PE was found besides 100% certainty. Moreover, the abovementioned study (Leclercq and Charras, 2026) systematically varied the ISI before the distractor (i.e., through aging and non-aging distributions). Yet, even under conditions promoting suppression and proactive inhibition, the PE persisted, suggesting that the PE cannot be accounted for by the informative value of the distractors for the dot-probe task (see also Makovski, 2019, Exp 5–7). Further, we emphasize that even in the No-distractor condition, an easily noticeable event, namely, the offset of the fixation cross, could have served as a temporal anchor for predicting the timing of the memory test, and therefore, a temporal-anchor account cannot explain the PE. Finally, it was found that preparation not only facilitates motor responses but also enhances perceptual encoding that does not require a speeded response (Shoval and Makovski, 2025), and hence a timed response could not fully account for the PE.

Experiment 2 demonstrated the rigidity of the PE as the effect was stable over time, and dot-probe responses were consistently faster in the distractor group than the no-distractor group across all four blocks. The magnitude of this advantage did not diminish even as general task performance improved equivalently for both groups. This finding is similar to foreperiod-based RT advantages that remain robust across extended practice, as long as the warning signal and the stimulus stay reliable and constant (Han and Proctor, 2022, 2023). The present between-subjects design showed a similar pattern in the PE paradigm: differences in dot-probe RT between groups were not diminished with time as the predictability of the distractors remained constant. This non-habituating pattern further distinguishes the PE from the attentional white bear effect, which diminishes and can be reversed with extensive practice (Cunningham and Egeth, 2016).

The sustained readiness observed in this study could reflect two related but conceptually distinct sources (Lawrence and Klein, 2013; McCormick et al., 2018). The first is a tonic process: a stable, slowly varying background readiness that persists as long as the context remains predictable. The second is an endogenous mode of temporal attention that is generated by learned signal-target history and directed toward a specific anticipated moment rather than toward general arousal. The distinction matters because tonic and endogenous are not synonyms: tonic refers to the temporal profile of a state (sustained rather than cue-locked), whereas endogenous refers to its origin (internally generated from learned regularities rather than driven by external stimulus intensity). The block-stable PE fits both descriptors simultaneously: it is endogenous in that it requires reliable distractor expectancy to engage, and it is tonic in that once engaged, it does not reset between trials or blocks. Yet, unlike genuine tonic alertness, it produces a targeted, time-locked readiness for the anticipated event without raising the general processing baseline.

Experiment 3 tested the paradigm used to test the PE by replacing the standard onset-detection probe with an offset-detection task, addressing the possibility that previous findings relied on onset-specific advantages. Offset-probe reaction times showed no significant advantage, in contrast to the robust facilitation observed in previous studies. However, the BF in favor of the null was anecdotal, and a comparison with an onset-based similar experiment (Lindzen et al., 2025) did not show a reliable interaction between tasks and facilitation. Offset responses were also, on average, slower (approximately 110 ms) than onset responses, and this, in turn, could have masked a small effect. Nevertheless, despite the lack of strong statistical results, and the implication of an offset task is still ambiguous, we believe that the lack of a reliable effect in Experiment 3 suggests that PE is weak and difficult to detect under these conditions. Still, this point should be taken cautiously, and future research should directly compare onset and offset probes in a single experiment.

Previous studies demonstrated that the PE is robust and replicable across different stimuli (Chen et al., 2026), memory loads (Chen et al., 2023), individual differences (Lindzen et al., 2025), with both dot probes, auditory probes (Makovski, 2019, experiment 5), and letter-encoding probes (Shoval and Makovski, 2025). However, if one accepts Experiment 3 results as reflecting reduced PE for offsets, then the present results indicate that PE exhibits some task-level boundaries, and preparatory alertness appears tuned to specific task demands. This might be explained in two ways. The first concerns the attentional set established by the distractor context. Both the distractor and the onset probe are abrupt visual events, so the preparatory state built up through distractor expectancy may be tuned specifically toward the detection of onset-type transients. Thus, PE, and presumably more broadly, phasic alertness, boosts readiness specifically for onset transients, which suggests that temporal readiness does not generalize uniformly across all detection demands (Leclercq and Charras, 2026).

The second explanation relies primarily on exogenous attention, which is simply weaker for stimulus offsets than for onsets in an attended location (Theeuwes, 1991). According to this view, the PE may generalize in principle across probe types, but offset probes are intrinsically less effective at eliciting an exogenous attentional response, so any preparatory benefit is too weak to measure reliably. Moreover, offset detection may rely more on perceptual change detection or decision processes that are less sensitive to this alerting state (Carmel and Lamy, 2014; Lamy et al., 2015). Thus, offset tasks are ill-suited to capture PE and other phasic alertness effects. Future research should further examine which task-dependent processes tap (or not) to PE and phasic alertness, and test whether it generalizes to alternative behavioral, no-report measures (e.g., pupillometry) and neural indices of anticipatory attention.

Collectively, the three experiments substantially refine the characterization of the PE and help reconcile earlier characterizations of the PE as rigid and non-flexible (Makovski, 2019) with more recent demonstrations of context-dependent variability (Chen et al., 2023, 2026; Leclercq and Charras, 2026) and position the PE as a conditional but durable form of temporal preparation. Preparation requires complete distractor certainty; it does not scale with distractor probability and sustains its effect over time. This pattern parallels phasic alertness effects demonstrated by signal-warning cues, foreperiod-based preparation effect (Han and Proctor, 2022, 2023). The expression of this preparatory effect, however, might be task-selective: it is robustly captured by onset-detection probes but not as effectively by offset-detection probes.

To conclude, the present study shows that people prepare for the presentation of upcoming stimuli only when these appear under complete certainty. This effect is durable and can be long-lasting, yet this preparation effect is best captured by onset detection tasks. Thus, the PE is not fully mandatory and can be modulated by task demands and the observer’s expectations.

## Data Availability

The datasets presented in this study can be found in online repositories. The names of the repository/repositories and accession number(s) can be found in the article/supplementary material.

## Footnotes

1 In previous studies, we used a variable interval to avoid the distractors predicting the appearance of the test-probe. Due to a coding error, trials here were blocked by a fixed interval. This factor did not interact with any other effect and did not affect any conclusion.

## Author’s note

The data, materials, and analyses are available at OSF: https://osf.io/8a3ec. This study was funded by the Open University research authority and the Israel Science Foundation (1274/21). The study was approved by the Ethics Committee (#3308). The authors wish to acknowledge Roy Shoval, Naama Or Leibovitz, Nitay Duek, and Yuval Megidish for their help and support in data collection.
